# Transition from Delusional Jealousy to Obsessive Symptoms : A Lacanian Psychopathological Perspective

**DOI:** 10.1192/j.eurpsy.2026.11801

**Published:** 2026-07-31

**Authors:** M. Bouchendira, H. Nefzi, H. Ghabi, D. Njah, R. Kammoun, M. El Karoui, F. Ellouze

**Affiliations:** 1 Emergencies and consultations; 2 Ibn Jazzar “G”, RAZI hospital, Manouba, Tunisia

## Abstract

**Introduction:**

Delusional jealousy is classically associated with paranoid personality traits and can mask unconscious conflicts. Transitions from psychotic delusional forms to neurotic obsessive forms are rarely documented, providing a unique window into the dynamics of defense mechanisms and symptom evolution.

**Objectives:**

To illustrate the psychopathological evolution from delusional jealousy to obsessional homosexual symptoms in a patient, highlighting Lacanian mechanisms and implications for clinical management.

**Methods:**

This study is based on the 17-year follow-up of a 53-year-old man with paranoid personality traits, documented through repeated clinical interviews and medical records from the Department “G” of Psychiatry at RAZI Hospital.

**Results:**

The patient initially presented at the age of 36 with persistent delusional jealousy, characterized by recurrent accusations of infidelity directed at his spouse. From a Lacanian perspective, this delusional construction can be understood as the projection of an unconscious homosexual desire, whereby the subject attributes to the partner the desire he cannot acknowledge in himself, externalizing an internal conflict. Defense mechanisms such as projection and splitting were predominant, allowing the psychotic structure to maintain coherence.

After years of clinical evolution, the delusional jealousy disappeared under treatment and he maintained a stable relationship with his wife and kids with no relapses and no hospitalisations. At the age of 53 the patient developed intrusive obsessional thoughts with homosexual content. Unlike the former psychotic certainty, these new symptoms were recognized by the patient as intrusive and ego-dystonic, causing significant distress. This transition illustrates a shift from psychotic to neurotic defenses, where the unconscious conflict resurfaced in a less delusional but still symptomatic form; obsessions representing the return of the repressed at a neurotic level.

**Conclusions:**

This case highlights the dynamic interplay between psychotic and neurotic defense mechanisms, demonstrating that obsessive symptoms may emerge as the psychotic structure stabilizes or deactivates. Lacanian analysis provides a framework to understand this transition, suggesting that treatment should address not only symptoms but also the underlying psychodynamic mechanisms. Awareness of such transitions is crucial for long-term management and prognosis.

**Disclosure of Interest:**

None Declared

